# Supplementary material for: Antiferroelectric negative capacitance from a structural phase transition in zirconia
Source: Nat Commun. 2022 Mar 9;13:1228. doi: 10.1038/s41467-022-28860-1 (PMC8907358; doi:10.1038/s41467-022-28860-1)
Supplement: Supplementary file 1 — Supplementary Information [file 41467_2022_28860_MOESM1_ESM.pdf]

Supplementary Information: Antiferroelectric negative capacitance from a structural phase transition in zirconia

Supplementary Methods

Phenomenological model of antiferroelectric negative capacitance. According to Kittel's phenomenological model of antiferroelectricity[1–3], an antiferroelectric crystal can be represented by two sub-lattices with anti-parallel polarization vectors with magnitudes of P_a and P_b. The free energy density G can be expressed in terms of sub-lattice polarizations P_a and P_b as follows.

$$G = a(P_a^2 + P_b^2) + b(P_a^4 + P_b^4) + hP_aP_b - (P_a + P_b)E_a \tag{1}$$

Here, $a < 0$, $b > 0$ are anisotropy constants. $h > 0$, which represent the anti-polar coupling between the sub-lattices. The system can be conveniently described in terms of two state variables: the staggered polarization $Q = P_a - P_b$, which is often considered the true antiferroelectric order parameter, and the macroscopically measurable polarization $P = P_a + P_b$. Substituting $P_a = (P+Q)/2$ and $P_b = (P-Q)/2$ in Eq. (1), the free energy density of an antiferroelectric material, G, can be expressed as follows.

$$G = \frac{1}{2}(a + \frac{1}{2}h)P^2 + \frac{1}{2}(a - \frac{1}{2}h)Q^2 + \frac{1}{8}b(P^4 + Q^4 + 6P^2Q^2) - PE_a \tag{2}$$

Defining $\alpha = (a + h/2)/2$, $\beta = (a - h/2)/2$ and $\zeta = b/8$, the following equation is obtained.

$$G = \alpha P^2 + \beta Q^2 + \zeta(P^4 + Q^4 + 6P^2Q^2) - PE_a \tag{3}$$

Furthermore, $\beta = \lambda(T - T_C)$ and $\alpha = \lambda(T - T_C) + h/2$ where T and T_C are the temperature and the Curie temperature of the antiferroelectric, respectively and $\lambda > 0$. When $T_C - h/2\lambda < T < T_C$,

$\alpha > 0$ and $\beta < 0$, and the $P - E_a$ curve exhibits two non-overlapping hysteresis loops as shown in Fig. 1a. Minimizing G with respect to P and Q for a given E_a leads to the following relations.

$$\frac{\partial G}{\partial P} = 2\alpha P + 4\zeta P^3 + 12\zeta P Q^2 - E_a = 0 \tag{4}$$

$$\frac{\partial G}{\partial Q} = 2\beta Q + 4\zeta Q^3 + 12\zeta P^2 Q = 0 \tag{5}$$

Solving Eq. (5), the following relation between P and Q is obtained.

$$Q = \begin{cases} \pm\sqrt{-\frac{\beta}{2\zeta} - 3P^2}, \text{ when, } |P| < P_n \text{ (non-polar)} \\ 0, \text{ when, } |P| \geq P_n \text{ (polar)} \end{cases} \tag{6}$$

Here, $P_n = \sqrt{-\beta/(6\zeta)}$ which defines the P-boundary between the non-polar reference phase and the polar phase. Note that $Q \neq 0$ and $Q = 0$ represent the non-polar reference phase and the polar phase, respectively. Replacing Q with the expression in Eq. (6) in Eq. (1), G can be expressed in terms of P as follows.

$$G = \begin{cases} (\alpha - 3\beta)P^2 - 8\zeta P^4 - PE_a - \frac{\beta^2}{4\zeta}, \text{ when, } |P| < P_n \text{ (non-polar)} \\ \alpha P^2 + \zeta P^4 - PE_a, \text{ when, } |P| \geq P_n \text{ (polar)} \end{cases} \tag{7}$$

Eq. (7) is used to generate the antiferroelectric energy landscapes at $E_a = 0$ and $E_a = \pm E_1$ in Fig. 1b-d. $\partial^2 G/\partial P^2$ is calculated as follows.

$$\frac{\partial^2 G}{\partial P^2} = \begin{cases} 2(\alpha - 3\beta) - 96\zeta P^2, \text{ when, } |P| < P_n \text{ (non-polar)} \\ 2\alpha + 12\zeta P^2, \text{ when, } |P| \geq P_n \text{ (polar)} \end{cases} \tag{8}$$

According to Eq. (8), $\partial^2 G/\partial P^2 < 0$ when $P_{nn} \leq |P| \leq P_n$ with $P_{nn} = \sqrt{(\alpha - 3\beta)/(48\zeta)}$. This range of P corresponds the segments BC and B'C' shown in Fig. 1. Combining Eq. (4) and (6),

the following relation between E_a and P can be obtained.

$$E_a = \begin{cases} 2(\alpha - 3\beta)P - 32\zeta P^3, \text{ when, } |P| < P_n \text{ (non-polar)} \\ \\ 2\alpha P + 4\zeta P^3, \text{ when, } |P| \geq P_n \text{ (polar)} \end{cases} \tag{9}$$

Note in Eq. (9) that $(\alpha - 3\beta) > 0$ since $\alpha > 0$ and $\beta < 0$ for which the $P - E_a$ curve has positive slope at and around $P = 0$ in the non-polar reference phase. On the other hand, it can be shown that $\partial E_a/\partial P < 0$, when $P_{nn} \leq |P| \leq P_n$ which results in the negative capacitance segments BC and B'C' in Fig. 1a.

Modeling of antiferroelectric negative capacitance field-effect transistors A well-designed antiferroelectric negative capacitance field-effect transistor (NCFET) can be obtained by the shifting $P - E_a$ curve through work-function engineering as experimentally demonstrated in Ref. [4] and by using only one of the two negative capacitance regions. In that case, the NCFET will be in a positive capacitance state in its off-state condition, and will provide a boost in the on-current for a given off-current due to the antiferroelectric negative capacitance. We modeled an antiferroelectric NCFET in which a 1.8 nm antiferroelectric layer is used in the gate dielectric stack of a 15 nm node baseline FinFET. The industry standard 15 nm Berkeley SPICE Insulated-Gate-FET Model: Common Multi Gate (BSIM-CMG) model is used to simulate the baseline FinFET. Following are the baseline FinFET device parameters of the Nangate FreePDK15 Open Cell Library (15 nm technology) used: fin height H_{fin} = 42 nm, fin thickness T_{fin} = 7 nm and gate length L_G = 20 nm. The antiferroelectric oxide layer is modeled using the relation: $E_a(Q) = \alpha_{AF}(Q - Q_\circ) + \beta_{AF}(Q - Q_\circ)^3 - E_{bias}$, where E_a is the electric field in the antiferroelectric layer, Q is the polarization/surface charge density, α_{AF} and β_{AF} are anisotropy constants

of the antiferroelectric, and E_{bias} is a built in electric field obtained by work-function engineering. The anisotropy constants are related to the antiferroelectric polarization $P_{\circ}^{AF}$ and the width of the antiferroelectric hysteresis loop E_H by the relations: $\alpha_{AF} = -3\sqrt{3}/2 \times (E_H/P_{\circ}^{AF})$ and $\beta_{AF} = 6\sqrt{3} \times E_H/(P_{\circ}^{AF})^3$. For our simulations, we first fitted $P_{\circ}^{AF} = 15$ μC cm^{-2} and $E_H = 1.8$ MV cm^{-1} to the experimental data for 10 nm ZrO_2 as shown in Supplementary Fig. 11. However, recently, it was found that the distance between the critical fields of the tetragonal-to-orthorhombic and the orthorhombic-to-tetragonal phase transitions increases with decreasing ZrO_2 thickness, due to an increase in the tetragonality of the unit cell.[5] To accommodate for this increased tetragonality expected for a 1.8 nm thick ZrO_2 film in an NCFET, we increased E_H to 3 MV cm^{-1} for the simulation. $Q_{\circ} = A_G \times P_{\circ}^{AF}/2$ where A_G is the FinFET gate area. We implemented a compact model of AFE NCFET that self consistently solves the BSIM-CMG model of the baseline FinFET and the antiferroelectric $E_a(Q)$ relation. The off-current was set to 10 nA for the antiferroelectric NCFET and the baseline FinFET.

Supplementary Fig. 13a shows the drain current I_D-gate voltage V_{GS} characteristics of an n-type antiferroelectric NCFET and the equivalent n-type baseline FinFET at a drain voltage V_{DS} = 0.8 V, which is the nominal voltage of the 15 nm technology node. At the off-state and in the subthreshold region, the antiferroelectric layer does not provide any improvement in the subthreshold swing as shown in Extended Data. Fig. 13b; however, it enters the negative capacitance region at much higher current levels for which it provides a significant boost in the on-current. Using a Synopsys HSPICE simulation tool, we also simulated a fan-out 4 (FO4) inverter (an inverter driving four identical inverters) based on antiferroelectric NCFETs at the power supply voltage

V_{DD}=0.8 V and 0.46 V and compared its characteristics with that based on the baseline FET operating at V_{DD}=0.8 V. We note in Table 1 that the antiferroelectric NCFET inverter operating at $V_{DD} = 0.46$ V provides the same performance ($i.e.$ delay) as that of the baseline FinFET inverter at $V_{DD} = 0.8$ V. As such, in this case, an iso-performance power reduction of 41% is obtained by using the antiferroelectric NCFET.

Table 1: **Power and performance comparison between an antiferroelectric (AFE) negative capacitance field-effect transistor (NCFET) and the equivalent baseline transistor.** The AFE NCFET operating at a power supply voltage V_{DD} = 0.46 V achieves the same performance as that of the baseline transistor at V_{DD} = 0.8 V, thereby reducing the power dissipation by 41% in this particular simulation. The AFE NCFET simulations was performed by implementing a compact model based on the industry standard 15 nm Berkeley SPICE Insulated-Gate-FET Model: Common Multi Gate (BSIM-CMG) model.

	Baseline FinFET		Antiferroelectric NCFET	
V_{DD} (V)	0.80	0.46	0.80	0.46
Average Delay (ps)	6.155	10.800	3.989	6.122
Total Power (fW)	4.110	1.153	6.570	2.425

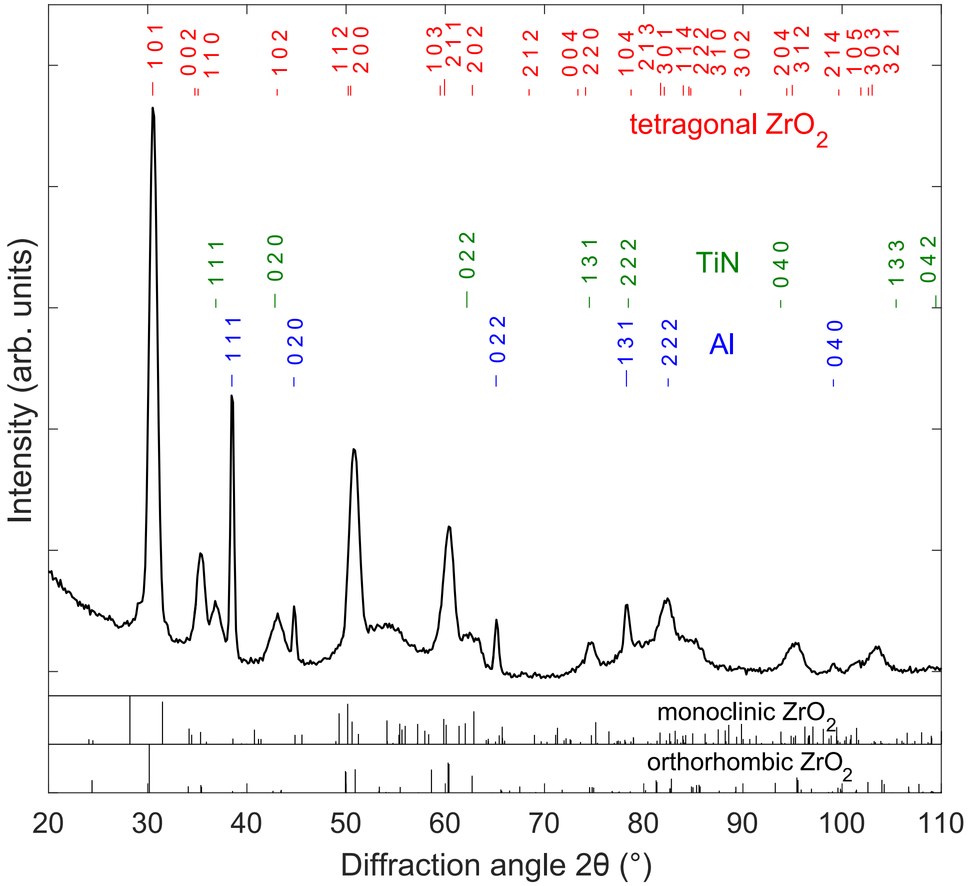

Supplementary Fig. 1: **Grazing-incidence X-ray diffraction measurement of ZrO_2.** Diffraction patterns and their indices for tetragonal ZrO_2 as well as TiN an Al phases are marked in the figure. For comparison, the position of the diffraction peaks of orthorhombic ZrO_2 and monoclinic ZrO_2 are indicated at the bottom. The Bragg peaks for ZrO_2 match well with those of the tetragonal structure. No fractions of the orthorhombic and monoclinic phase were observed in our samples based on X-ray diffraction data, which is consistent with scanning transmission electron microscopy and nanobeam electron diffraction results (see Fig. 2b and Supplementary Fig. 3).

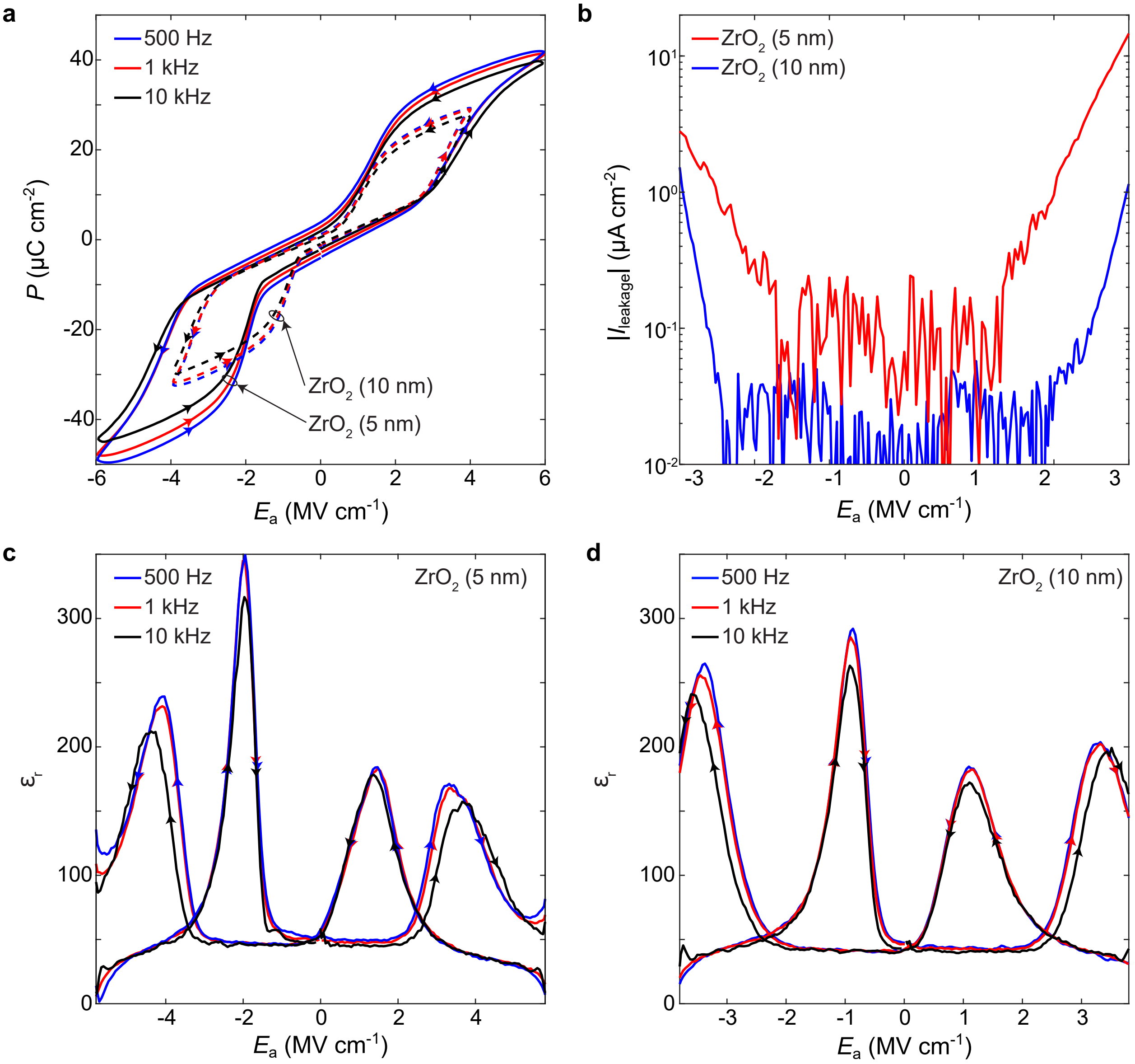

Supplementary Fig. 2: **Electric characterization of stand-alone ZrO_2 capacitors. a** Polarization P - electric field E_a loops of 5 nm (solid line) and 10 nm (dashed line) ZrO_2 capacitors at 500 Hz, 1 kHz, and 10 kHz. **b** Leakage current $I_{leakage}$ - electric field plot for 10 nm (blue color) and 5 nm (red color) ZrO_2 capacitors. **c, d** Dielectric constant ε_r - electric field curve of 5 nm and 10 nm ZrO_2 capacitors at 500 Hz, 1 kHz, and 10 kHz, respectively. The dielectric constant is obtained by differentiating the polarization P - electric field E_a plot in **a**.

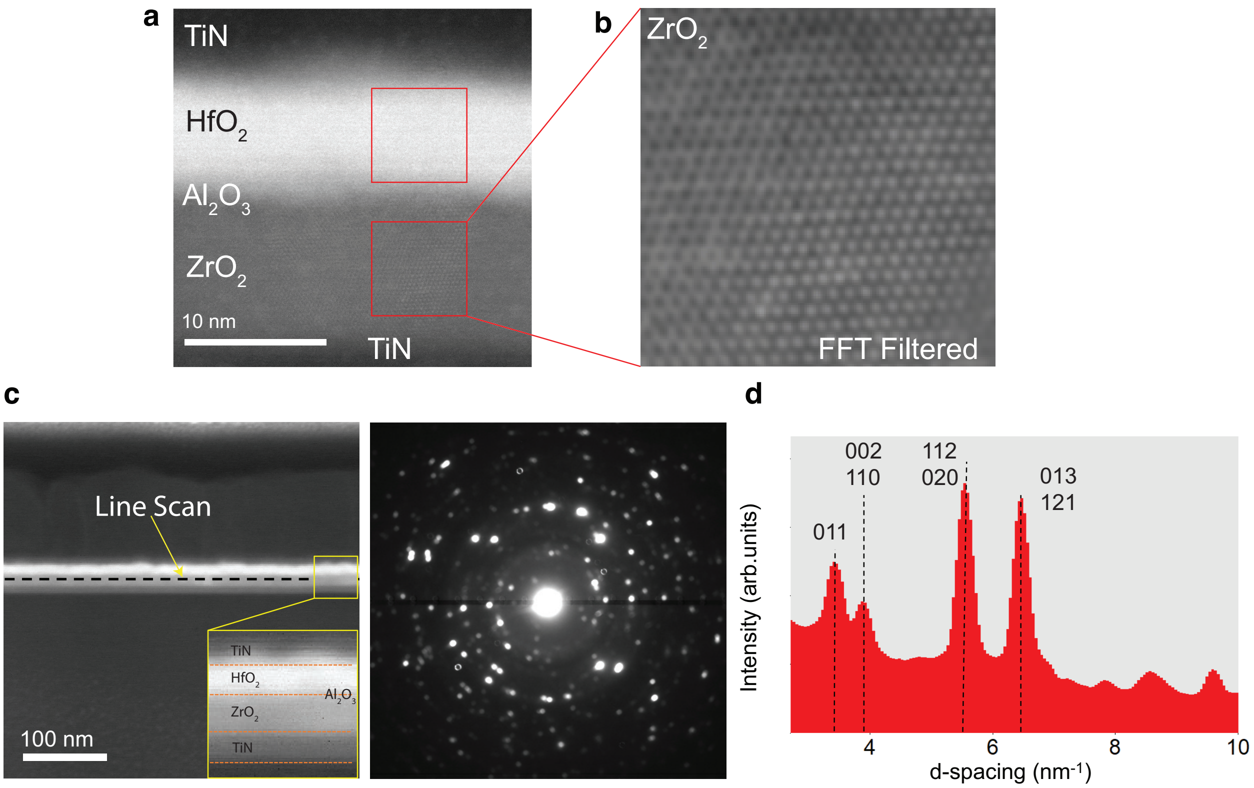

Supplementary Fig. 3: **Phase determination of ZrO_2 in dielectric-antiferroelectric heterostructures.** **a** High magnification high angle annular dark field (HAADF) scanning transmission electron microscopy (STEM) image of the cross-section of the same TiN/HfO_2/Al_2O_3/ZrO_2/TiN heterostructure shown in Fig. 2b. Fast Fourier transform (FFT) patterns shown in Fig. 2b were taken from regions under the red squares in the HfO_2 and ZrO_2 layers. **b** The enlarged, FFT filtered image taken from the region in ZrO_2. **c** HAADF-STEM image of the heterostructure with a line scan region along which nanobeam diffraction patterns were collected highlighted by dashed black lines. An enlarged image taken from the yellow region shows the TiN, HfO_2, ZrO_2 and TiN layers with dashed red lines highlighting the interfaces. A total of 200 nanobeam diffraction patterns were collected, with 2 nm spacing between each pattern. The diffraction pattern matches with that of tetragonal ZrO_2 well. **d** Summed radial intensity profile extracted from **b**.... *(continues to next page)*

Supplementary Fig. 3: *(continues from previous page)* The peak at about 3.4 nm^{-1} corresponds to the reflection from {011}. The peak at 3.9 nm^{-1} originates from reflections of {002} and {110} where we can not resolve those peaks due to a poor resolution of electron beam diffraction. The peak at 5.5 nm^{-1} originates from {112} and {020} and 6.5 nm^{-1} from {013} and {121}. The peaks were found to match well with that from a tetragonal structure, not to those of monoclinic and orthorhombic ones.

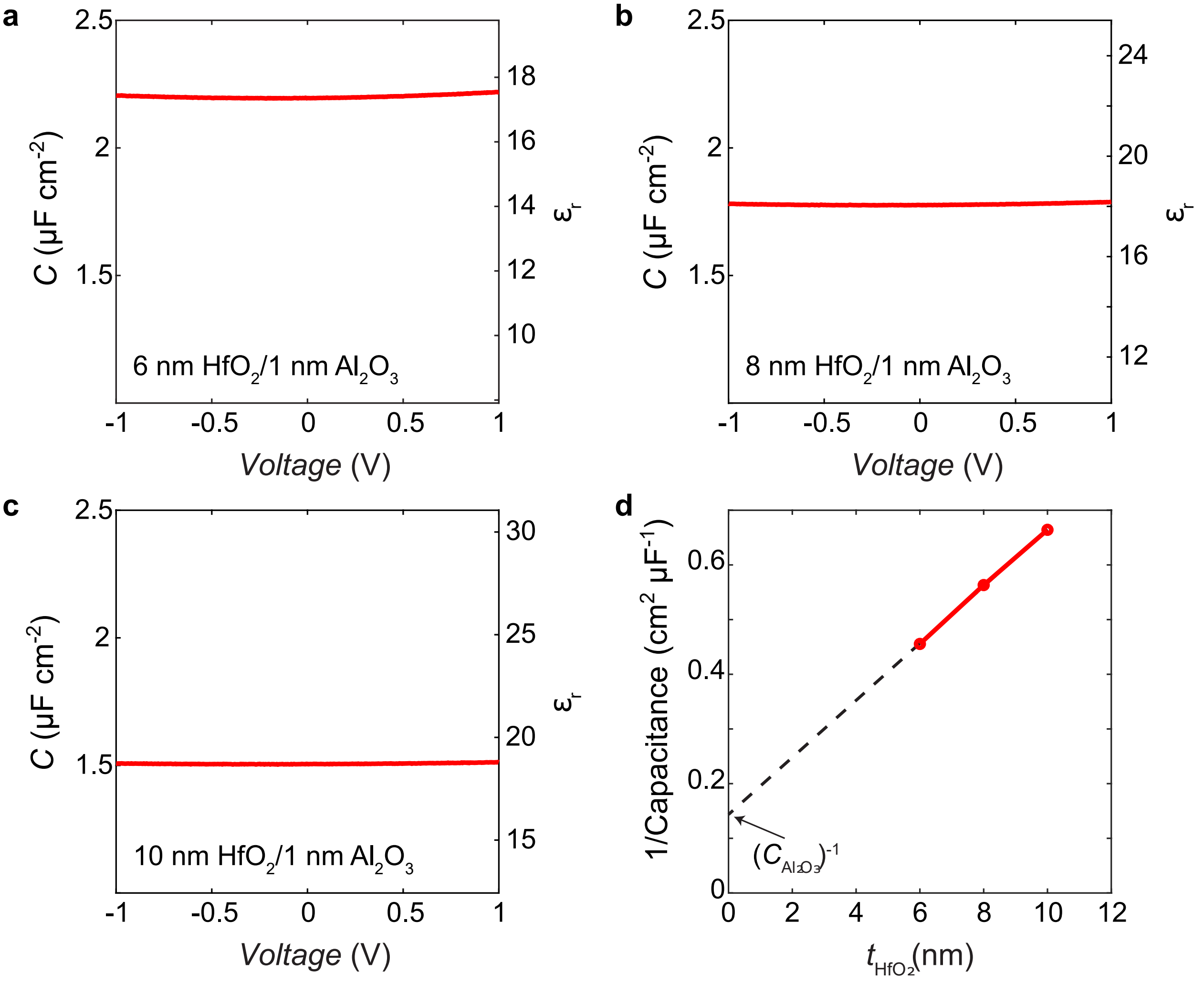

Supplementary Fig. 4: **Electrical characterization of HfO_2/Al_2O_3 dielectric stacks. a, b, c** Capacitance C and dielectric constant ε_r as a function of voltage across HfO_2/Al_2O_3(1 nm) capacitors where the thicknesses of HfO_2 are 6 nm (**a**), 8 nm (**b**), and 10 nm (**c**). **d** Inverse capacitance of the dielectric stacks as a function of HfO_2 thickness. The slope of the curve is $(\epsilon_{\circ}\epsilon_{HfO_2})^{-1}$, and the y-axis intercept is $C_{Al_2O_3}^{-1}$. $\epsilon_{\circ}$, ϵ_{HfO_2} and $C_{Al_2O_3}$ are the vacuum permittivity, the relative dielectric constant of HfO_2 and the capacitance of $\sim$1 nm Al_2O_3 in our dielectric stacks, respectively. The dielectric constants of HfO_2 and Al_2O_3 are extracted to be $\sim$21.5 and $\sim$8, respectively.

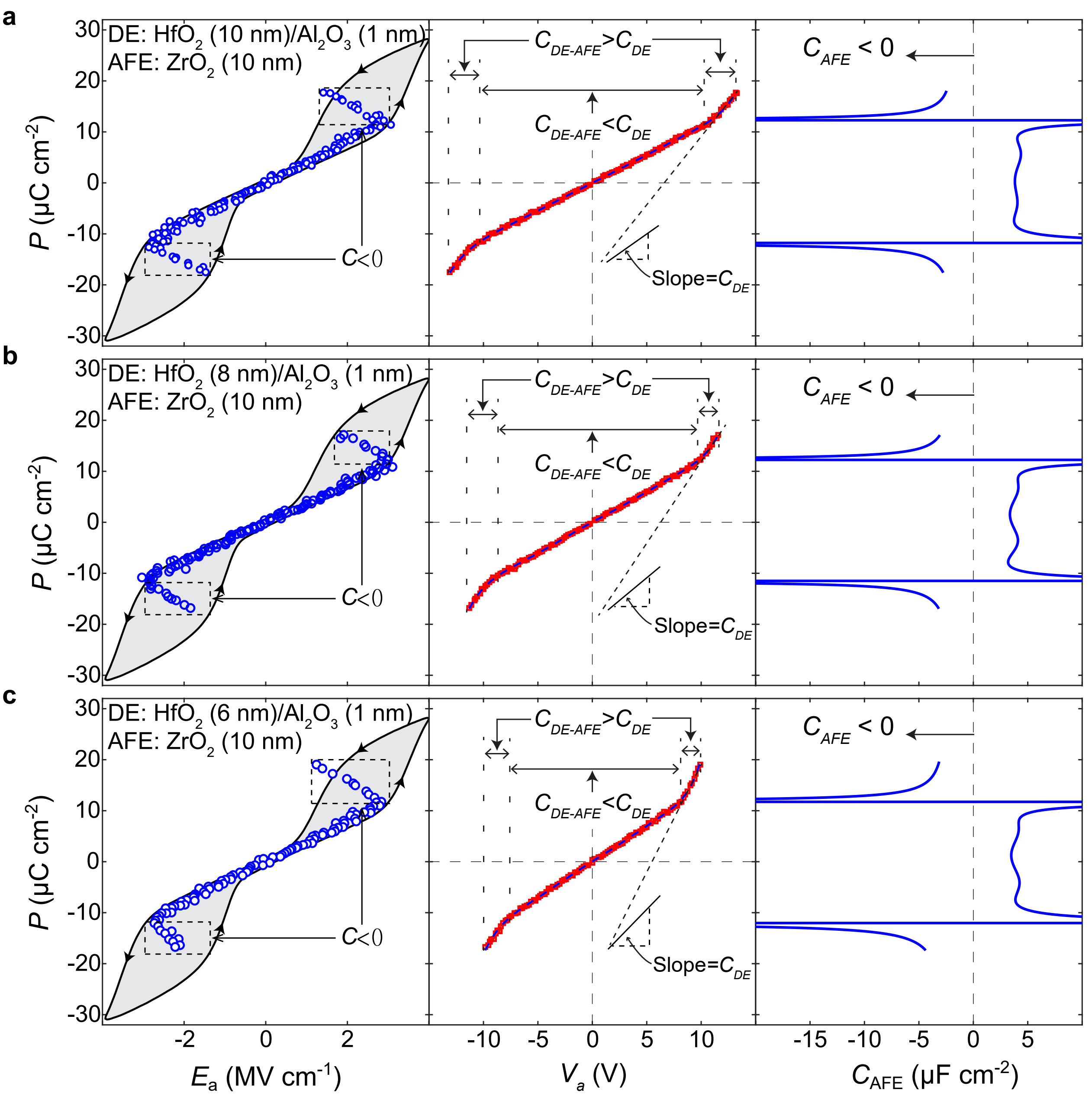

Supplementary Fig. 5: **Dielectric layer thickness dependence of antiferroelectric negative capacitance.** Polarization P as functions of electric field E_a of the ZrO_2 layer, maximum voltage across the dielectric/antiferroelectric capacitor V_a, antiferroelectric capacitance C_{AFE} measured in HfO_2/Al_2O_3(1 nm)/ZrO_2(10 nm) heterostructure capacitors with HfO_2 thicknesses of 10 nm (**a**), 8 nm (**b**), and 6 nm (**c**). C_{DE} and C_{DE-AFE} are the dielectric and total capacitance, respectively.

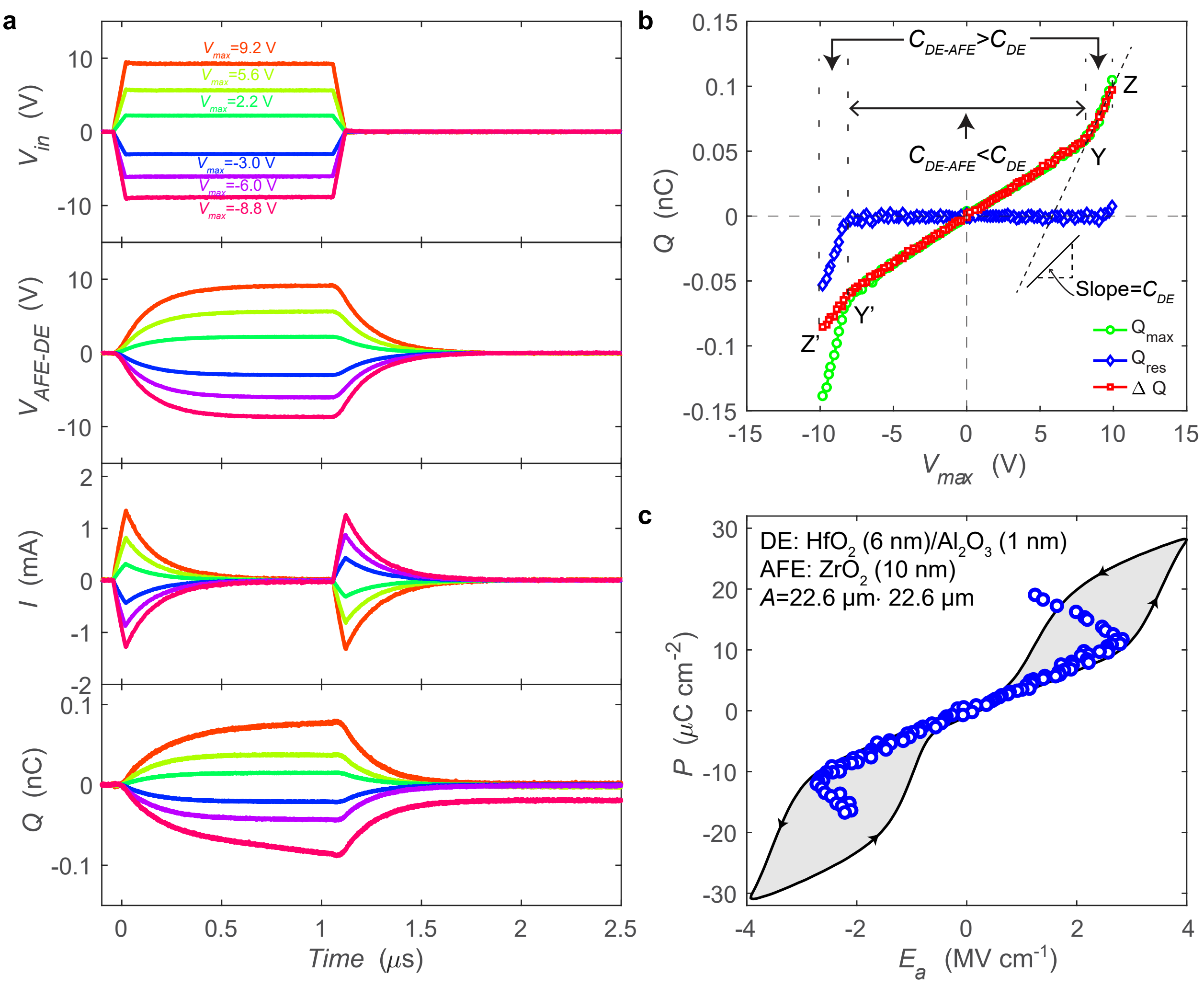

Supplementary Fig. 6: **Electrical measurement of a HfO_2(6 nm)/Al_2O_3(1 nm)/ZrO_2(10 nm) capacitor.** **a** Time domain waveforms of input voltage V_{in}, voltage across the heterostructure V_{DE-AFE}, current through the heterostructure I, and integrated charges Q. **b** Maximum charge Q_{max}, residual charge Q_{res}, and their difference ΔQ as a function of maximum voltage across the heterostructure V_a. **c** Extracted polarization P - electric field E_a of the ZrO_2 layer.

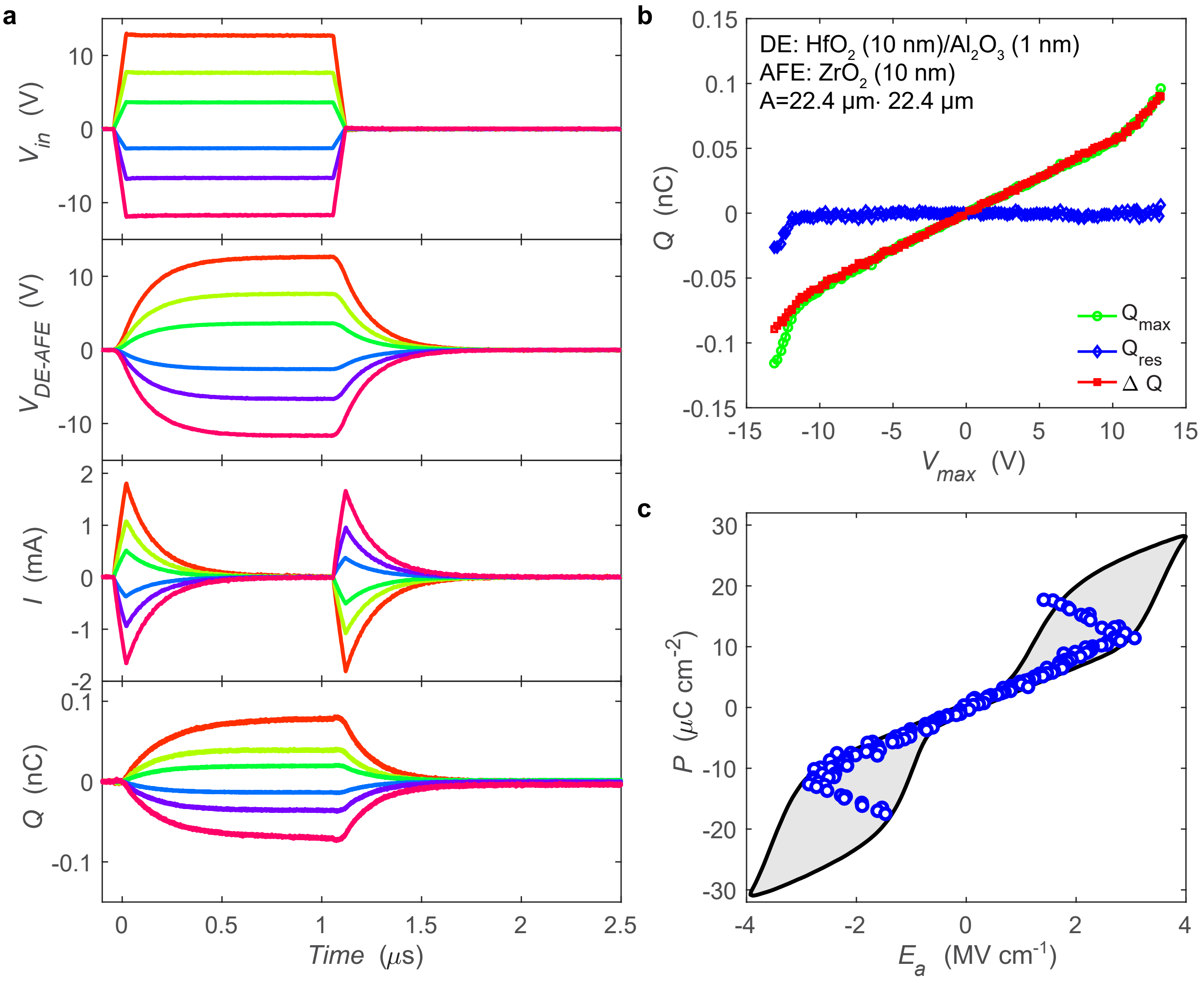

Supplementary Fig. 7: **Electrical measurement of a HfO_2(10 nm)/Al_2O_3(1 nm)/ZrO_2(10 nm) capacitor.** **a** Time domain waveforms of input voltage V_{in}, voltage across the heterostructure V_{DE-AFE}, current through the heterostructure I, and integrated charges Q. **b** Maximum charge Q_{max}, residual charge Q_{res}, and their difference ΔQ as a function of maximum voltage across the heterostructure V_a. **c** Extracted polarization P - electric field E_a of the ZrO_2 layer.

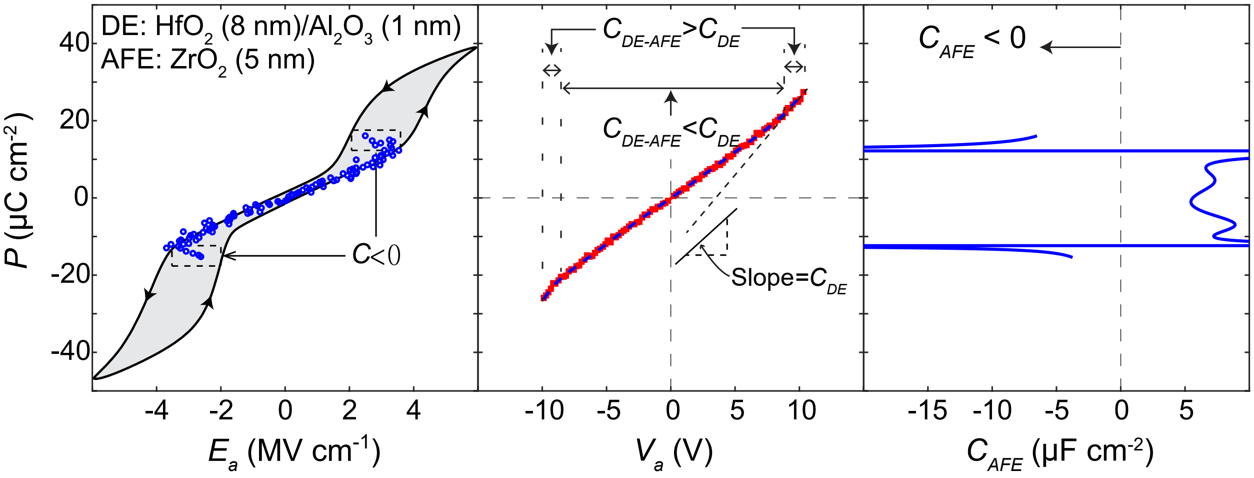

Supplementary Fig. 8: **Antiferroelectric layer thickness dependence on antiferroelectric negative capacitance.** Polarization P as functions of electric field E_a of the ZrO_2 layer, maximum voltage across the dielectric/antiferroelectric capacitor V_a, antiferroelectric capacitance C_{AFE} measured in a HfO_2(8 nm)/Al_2O_3(1 nm)/ZrO_2(5 nm) heterostructure. C_{DE} and C_{DE-AFE} are the dielectric and total capacitance, respectively.

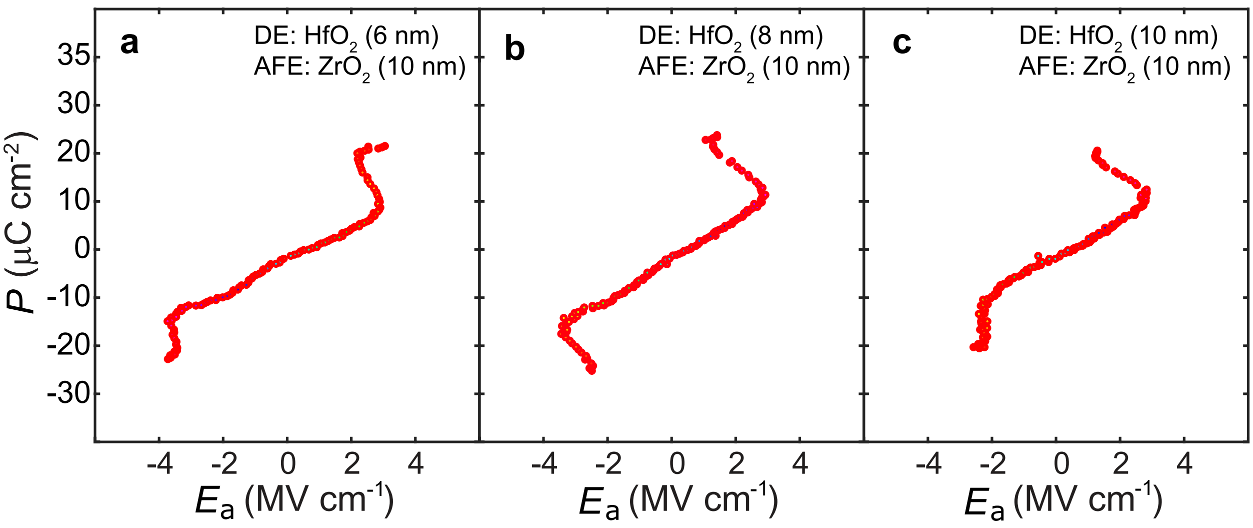

Supplementary Fig. 9: **Observation of the high-field positive capacitance branch.** Polarization P as a function of electric field E_a in the ZrO_2 layer of antiferroelectric/dielectric capacitors with 10 nm ZrO_2 and 6 nm (**a**), 8 nm (**b**) and 10 nm (**c**) HfO_2. Data was extracted from pulsed voltage measurements (pulse width 1μs) analogous to Supplementary Fig. 5-7.

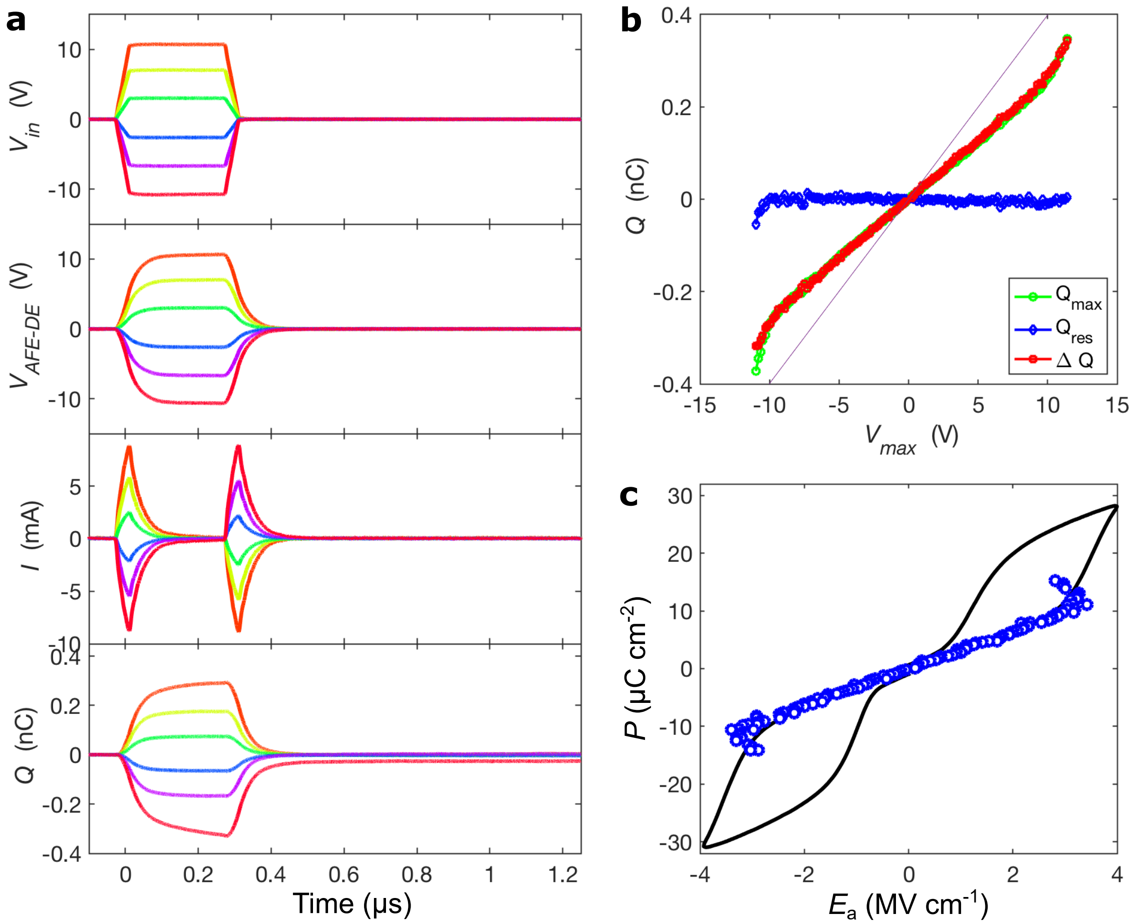

Supplementary Fig. 10: **Electrical measurement of a** HfO_2(8 nm)/Al_2O_3(1 nm)/ZrO_2(10 nm) capacitor with R = 560 Ω. **a** Time domain waveforms of input voltage V_{in}, voltage across the heterostructure V_{DE-AFE}, current through the heterostructure I, and integrated charges Q. **b** Maximum charge Q_{max}, residual charge Q_{res}, and their difference ΔQ as a function of maximum voltage across the heterostructure V_{max}. **c** Extracted polarization P - electric field E_a of the ZrO_2 layer.

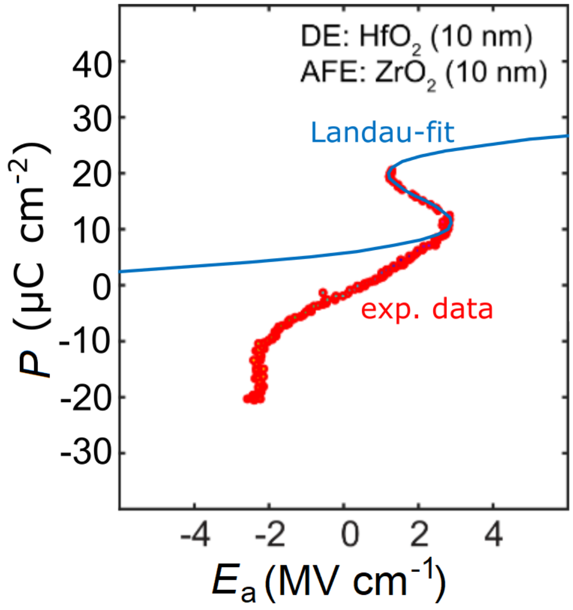

Supplementary Fig. 11: **Landau fitting of one of the negative capacitance regions.** Polarization P as a function of electric field E_a in the antiferroelectric ZrO_2 layer. Red dots show the data from short pulsed voltage measurements on a HfO_2(10 nm)/ZrO_2(10 nm) capacitor. The blue line shows the best fit of of one of the negative capacitance regions using a simple Landau model as described in the Supplementary Methods.

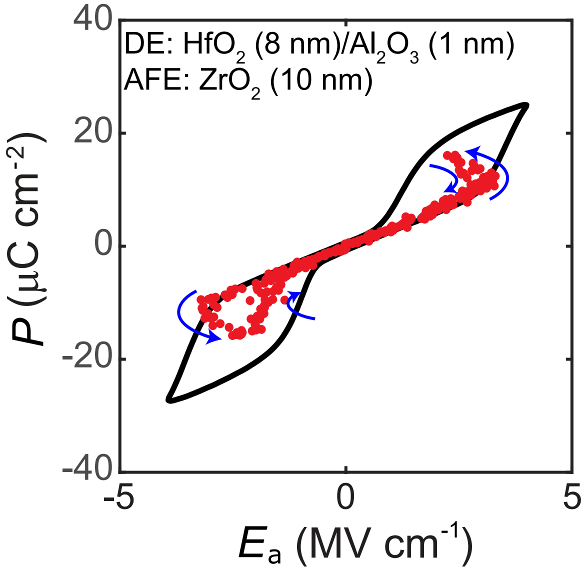

Supplementary Fig. 12: **Pulsed voltage hysteresis measurement.** Polarization P as a function of electric field E_a in the antiferroelectric ZrO_2 layer. The voltage pulse amplitude was first increased from 0 V to 11 V and then back to 0 V to -11 V and back to 0 V. Very small hysteresis is observed for positive applied voltages, since the leakage and charge trapping is small (Q_{res} is low). For negative applied voltages the hysteresis is large due to significant leakage current and subsequent charge trapping (Q_{res} is high).

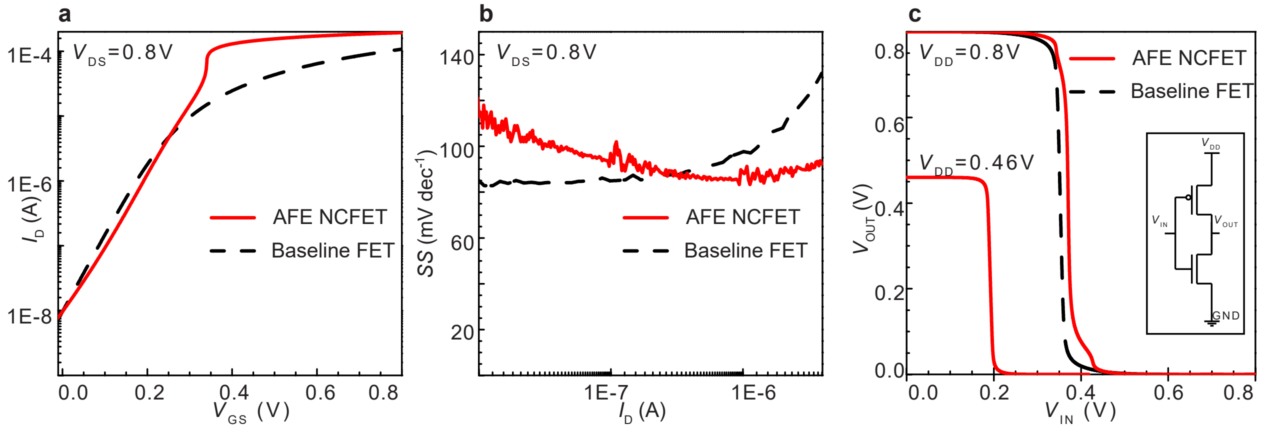

Supplementary Fig. 13: **Modeling of an antiferroelectric (AFE) negative capacitance field-effect transistor (NCFET). a,b** The drain current I_D vs. gate voltage V_{GS} characteristics (**a**) and the subthreshold swing (SS) vs. I_D characteristics of an n-type AFE NCFET and the equivalent n-type baseline FinFET at a drain voltage V_{DS} = 0.8 V. **c** The voltage transfer characteristics of a fan-out 4 (FO4) inverter based on AFE NCFET operating at power supply voltages V_{DD} = 0.8 V and 0.46 V and a FO4 inverter based on the equivalent baseline transistor at V_{DD} = 0.8 V. V_{IN} and V_{OUT} are the inverter input and output voltage, respectively. The details of the simulation framework is provided in Supplementary Methods. The power-performance comparison between the AFE NCFET and the equivalent baseline transistor based FO4 inverters is provided in Supplementary Table 1, which shows that the AFE NCFET is operating at a power supply voltage V_{DD} = 0.46 V and achieves the same performance as that of the baseline transistor at V_{DD} = 0.8 V, thereby reducing the power dissipation by 41%. The AFE NCFET simulations were performed by implementing a compact model based on the industry standard 15 nm Berkeley SPICE Insulated-Gate-FET Model: Common Multi Gate (BSIM-CMG) model.

Supplementary References

1. Kittel, C. Theory of antiferroelectric crystals. *Phys. Rev.* **82**, 729–732 (1951).

2. Okada, K. Phenomenological theory of antiferroelectric transition. i. second-order transition. *J. Physical Soc. Japan* **27**, 420–428 (1969).

3. Cross, L. E. Antiferroelectric-ferroelectric switching in a simple "Kittel" antiferroelectric. *Journal of the Physical Society of Japan* **23**, 77–82 (1967).

4. Pešić, M., Hoffmann, M., Richter, C., Mikolajick, T. & Schroeder, U. Nonvolatile random access memory and energy storage based on antiferroelectric like hysteresis in ZrO_2. *Adv. Func. Mater.* **26**, 7486–7494 (2016).

5. Tasneem, N. *et al.* A Janovec-Kay-Dunn-Like Behavior at Thickness Scaling in Ultra-Thin Antiferroelectric ZrO_2 Films. *Advanced Electronic Materials* **7**, 2100485 (2021).
