# Supplementary material for: Antiferroelectric negative capacitance from a structural phase transition in zirconia
Source: Nat Commun. 2022 Mar 9;13:1228. doi: 10.1038/s41467-022-28860-1 (PMC8907358; doi:10.1038/s41467-022-28860-1)
Supplement: Supplementary file 3 — Description of Additional Supplementary Files [file 41467_2022_28860_MOESM3_ESM.docx]

**Inventory of Supporting Information**
Supplementary Methods

Supplementary Table 1

Supplementary Fig. 1

Supplementary Fig. 2

Supplementary Fig. 3

Supplementary Fig. 4

Supplementary Fig. 5

Supplementary Fig. 6

Supplementary Fig. 7

Supplementary Fig. 8

Supplementary Fig. 9

Supplementary Fig. 10

Supplementary Fig. 11

Supplementary Fig. 12

Supplementary Fig. 13

Supplementary References
